# Evaluation of Real-Time PPP-Based Tide Measurement Using IGS Real-Time Service

**DOI:** 10.3390/s20102968

**Published:** 2020-05-24

**Authors:** Mingwei Di, Anmin Zhang, Bofeng Guo, Jiali Zhang, Rongxia Liu, Mengyuan Li

**Affiliations:** 1 School of Marine Science and Technology, Tianjin University, No. 92 Weijin Road, Nankai District, Tianjin 300072, China; dimingwei_marine@tju.edu.cn (M.D.); zhanganmin@sina.com (A.Z.); zh_jiali@yeah.net (J.Z.); liurongxia@tju.edu.cn (R.L.); limengyuan_tju@sina.com (M.L.); 2 Tianjin Port Environmental Monitoring Engineering Center, Tianjin 300072, China; 3 First Monitoring and Application Center, China Earthquake Administration, 7 Naihuo Road, Tianjin 300180, China; 4 Shandong Provincial Key Laboratory of Water and Soil Conservation and Environmental Protection, College of Resources and Environment, Linyi University, Linyi 276000, China

**Keywords:** GPS, real-time, tide measurement, precise point positioning, robust Vondrak filter

## Abstract

Tide data plays a key role in many marine scientific research fields such as seafloor topography measurement and navigation safety. To obtain reliable tide data, various methods have been proposed, e.g., tide station measurement, satellite altimeter measurement, and differential global positioning system (GPS) buoy measurement. However, these methods suffer from the limitation that continuous observations at different areas might not be always available. In order to provide high-precision as well as continuous real-time tide data, we propose a method based on real-time precise point positioning (RT-PPP) by using International GNSS Service (IGS) real-time service (RTS) products. Firstly, compared with the IGS final products, the accuracy of the RTS satellite orbit and clock is evaluated. Secondly, the positioning performance of RT-PPP is compared with the IGS ultra-fast products. Finally, a robust Vondrak filter is proposed to eliminate the influence of high-frequency noise and errors and to obtain tide results. Experimental results show that three-dimensional (3D) accuracy of the RTS orbit is better than 0.05 m, and also has 0.22 ns less clock bias. An improvement of 60% is achieved for positioning accuracy using RTS products compared to IGS ultra-fast products. Compared with the post-processing PPP method, the double difference (DD) method and tide gauge data, the root mean square (RMS) values of RT-PPP tide are 0.090, 0.194 and 0.167 m, respectively.

## 1. Introduction

Tide data provides rich information that facilitates marine scientific research such as seafloor topography and navigation safety. Tide gauge stations traditionally record tide measurements to obtain geophysical and oceanographic information [1]. Through long-term continuous observation of a certain sea areas, researchers can analyze the change of the sea level [1,2,3,4], study tide models [5,6] and investigate and forecast extreme sea level events such as hurricane storm surge and tsunami [2,7,8,9]. Tide data measured by tide gauges can achieve high precision, however, it is limited to coastal areas near tide gauge stations, and it is difficult to build tide gauges in open sea areas far away from the shore.

Since late 1992, high-quality satellite altimeters such as TOPEX/Poseidon, Jason-1 and OSTM/Jason-2 have provided near-global measurement of sea levels from which sea-level rise can be estimated. Altimeter records are used in monitoring changes in coastal sea levels and investigating tide behaviors [5,6,9,10,11,12,13,14]. When comparing sea surface heights obtained by altimetry and tide gauges nearby, there is a RMS values in the range of 0.08–0.89 m [14]. Satellite altimeters can meet the requirement of large-scale sea level observation and tide measurement. However, the spatial resolution, coupled with the several-days-long repeat orbit of satellite, is too coarse to monitor the evolution of ocean features.

In recent years, the global positioning system (GPS) has been experiencing dramatic changes. GPS is now suitable for all-weather, real-time and high-precision positioning, and thus widely used in positioning, navigation and timing applications, including oceanography. For example, it has been used for monitoring marine surface heights and assessing oceanographic models.

Ever since the proposition of the global navigation satellite system (GNSS) reflection (GNSS-R) measurement [15], some scholars have used GNSS reflection signals to monitor sea levels, such as GNSS-based tide gauge on land [16,17,18,19], airborne and eventually space-borne receivers [20,21,22]. Yet land-based receivers can only measure the sea surface within a certain distance from the coast, and the range is limited by the height of the GNSS tide gauge. The airborne and spaceborne receivers are usually strip measurements, which cannot provide continuous observation of a certain area.

In order to overcome the aforementioned shortcomings, some scholars use GPS buoys to measure the tide and monitor the sea level changes in the sea [13,23,24,25,26]. To obtain high-precision measurement data, they all use differential GPS techniques to reduce impacts of unmodelled satellite and receiver electronic biases and the mismodelling of GPS observations. However, the precision of differential kinematic positioning is baseline-length-dependent because errors at both sites decorrelate with the increasing baseline length. In addition, the performance of differential positioning techniques relies on the precision and accuracy of the reference station coordinates.

In the past decade, precise point positioning (PPP) has been developed in which undifferenced observations at only a single user station are processed to obtain decimeter-to-millimeter-level positioning accuracy when precise satellite orbits and clocks are provided [27]. Due to its excellent performance concerning efficiency, flexibility and positioning accuracy, the PPP approach plays a crucial role and is widely employed in such areas as precise positioning, timing, seismological and meteorological applications [28,29,30,31]. With the development of e-navigation and other technologies, real-time tide data and high-precision positioning information for vessels has become one of the research hot spots. Driven by recent advances in the International GNSS Service (IGS) real-time pilot project, the real-time precise satellite orbit and clock correction service is officially distributed online, to support real-time PPP (RT-PPP) services with global coverage.

This paper presents a tide measurement method based on RT-PPP. We first analyze the accuracy of the real-time service (RTS) products in both orbit and clock products. Then we compare the positioning results of RT-PPP with other two scenarios, including post-processing PPP using IGS final products and RT-PPP using IGS ultra-rapid (IGU) products. The descriptions of these two products are shown in Table 1 [32]. Affected by waves and even gross errors, the results obtained directly by the buoy cannot reflect the real tide information. Many scholars use low-pass filters to extract tide information [26,33], which are however less suitable for real-time measurement. Therefore, we propose a robust Vondrak filtering algorithm. Finally, the validation of tide measurement based on RT-PPP is evaluated by comparing with the post-processing PPP method, the double difference method and the nearby tide gauge data.

## 2. Materials and Methods

In order to meet the demand of RT-PPP, the International GNSS Service (IGS) established the Real-Time Working Group (RTWG) in 2001 and defined real-time service (RTS). In April 2013, the IGS officially launched the real-time service (RTS) to provide a precise orbit and clock correction service via the Radio Technical Commission for Maritime Services (RTCM) protocol for GPS and GLObal NAvigation Satellite System (GLONASS) [34,35]. Eight analysis centers have participated in the RTS: the BKG (Bundesamt für Kartographie und Geodäsie), the CNES (Centre National d’Etudes Spatiales), the DLR (Deutsches Zentrum für Luft- und Raumfahrt), the ESA/ESOC (European Space Agency’s Space Operations Centre), the GFZ (GeoForschungsZentrum), GMV (GMV Aerospace and Defense), NRCan (Natural Resources Canada) and WHU (Wuhan University). The RTS products are distributed as RTCM state-space representation (SSR) correction streams and broadcast over the internet using the networked transport of RTCM via internet protocol (NTRIP). They have been applied to earthquake monitoring and weather forecasting [36,37].

The real-time orbit and clock of broadcast ephemeris can be corrected using the RTS products in order to obtain precise products, which makes it possible to get a high-precision real-time position at different places. We use the kinematic PPP method to get the instantaneous sea level within the International Terrestrial Reference Frame 2014 (ITRF 2014). The buoy will move due to the influence of wind, waves and other factors, and the vertical coordinate will change in high frequency. Therefore, the instantaneous sea level obtained by GPS antenna cannot reflect the real tide information and some measures must be adopted to eliminate the influence of the above factors. 

**Figure 1:**
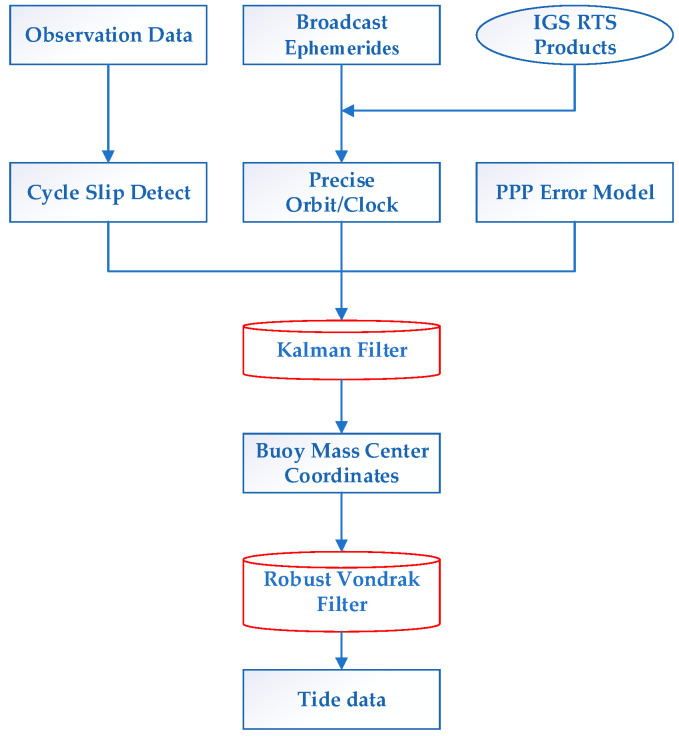
Flow chart of real-time tide measurement using real-time precise point positioning (RT-PPP) based on IGS real-time service (RTS) products.

The processing of the GPS measurement was carried out in three steps, i.e., real-time data receiving, post-processing calculation and filtering. In the first step, RTCM SSR correction streams were received in real time using BKG Ntrip Client (BNC) software. Meanwhile, observation data on the buoy was recorded. In the second step, RTKLIB software was used to obtain the instantaneous sea surface height within the ITRF 2014 based on the recorded RTCM SSR corrections. In the third step, a filtering algorithm was developed in matlab to obtain tide information. The specific real-time tide measurement method using RT-PPP is shown in Figure 1.

### 2.1. Real-Time Satellite Orbit and Clock Calculation

When calculating satellite coordinates, the RTS real-time corrections are given in the orbital coordinate system (radial, along-track and cross-track), which is not consistent with the broadcast ephemerides using the earth-centered-earth fixed (ECEF) coordinate system. Therefore, a conversion from the orbital coordinate system to ECEF system is needed. The calculation of the real-time satellite orbit is as follows.

Firstly, the real-time correction value of the current epoch *t* in the orbital coordinate system needs to be calculated [38]:

(1) $${\left[\begin{array}{c} d_{r}\\ d_{a}\\ d_{c} \end{array}\right]}_{t,orbit}={\left[\begin{array}{c} d_{r}\\ d_{a}\\ d_{c} \end{array}\right]}_{t_{0},orbit}+\left[\begin{array}{c} \dot{d_{r}}\\ \dot{d_{a}}\\ \dot{d_{c}} \end{array}\right]\left(t-t_{0}\right)$$

where $d_{r}$, $d_{a}$, $d_{c}$ are the radial, along-track and cross-track corrections, $t_{0}$ is the issue of date (IOD) and $\dot{d_{r}}$, $\dot{d_{a}}$ and $\dot{d_{c}}$ are the rate of the radial, along-track and cross-track corrections. Then, the rotation matrix R from the orbital coordinate system to the ECEF system and the correction values $d_{x}$, $d_{y}$ and $d_{z}$ in the ECEF system are calculated.

(2) $$R=\left[\frac{\dot{r}}{\left|\dot{r}\right|}\times \frac{r\times \dot{r}}{\left|r\times \dot{r}\right|}\;\frac{\dot{r}}{\left|\dot{r}\right|}\;\frac{r\times \dot{r}}{\left|r\times \dot{r}\right|}\;\right]$$

(3) $${\left[\begin{array}{c} d_{x}\\ d_{y}\\ d_{z} \end{array}\right]}_{t,ECEF}=R{\left[\begin{array}{c} d_{r}\\ d_{a}\\ d_{c} \end{array}\right]}_{t,orbit}$$

where r and $\dot{r}$ are the satellite position vector and satellite velocity vector calculated by broadcast ephemeris. Finally, the satellite coordinates $X_{0}$, $Y_{0}$ and $Z_{0}$ calculated by the broadcast ephemeris are corrected, and the corrected coordinates X, Y and Z are obtained.

(4) $${\left[\begin{array}{c} X\\ Y\\ Z \end{array}\right]}_{t,ECEF}={\left[\begin{array}{c} X_{0}\\ Y_{0}\\ Z_{0} \end{array}\right]}_{t,ECEF}-{\left[\begin{array}{c} d_{x}\\ d_{y}\\ d_{z} \end{array}\right]}_{t,ECEF}$$

Real-time satellite clock corrections are streamed in the form of polynomial coefficients $a_{0}$, $a_{1}$ and $a_{2}$. The clock correction dt and the precise satellite clock correction at epoch t are calculated by the following formula:

(5) $$dt=a_{0}+a_{1}\left(t-t_{0}\right)+a_{2}{\left(t-t_{0}\right)}^{2}$$

(6) $$t_{c}=t_{brd}-dt$$

where $t_{brd}$ is the clock bias at time t calculated from the broadcast ephemerides.

### 2.2. RT-PPP Model

The observation equations for undifferenced (UD) carrier phase L and pseudorange P, respectively, can be expressed as following [39]:

(7) $$\{\begin{array}{c} L_{r,j}^{s}=\rho_{r}^{s}+c\left(dt_{r}-dt^{s}\right)+\lambda_{j}\left(b_{r,j}-b_{j}^{s}\right)+\lambda_{j}N_{r,j}^{s}-I_{r,j}^{s}+T_{r}^{s}+\varepsilon_{r,j}^{s}\\ P_{r,j}^{s}=\rho_{r}^{s}+c\left(dt_{r}-dt^{s}\right)+\lambda_{j}\left(d_{r,j}-d_{j}^{s}\right)+I_{r,j}^{s}+T_{r}^{s}+e_{r,j}^{s}\; \end{array}$$

where s, r and j (j=1,2) refer to the satellite, receiver and carrier frequency, respectively; $\rho_{r}^{s}$ denotes the geometric distance between the phase centers of the satellite and receiver antennas at the signal transmitting and receiving time; $dt_{r}$ and $dt^{s}$ represent receiver clock bias and satellite clock bias; $\lambda_{j}$ is the wave-length; $b_{r,j}$ and $b_{j}^{s}$ are the receiver and satellite uncalibrated phase delay [40]; N is the integer ambiguity; $I_{r,j}^{s}$ is the ionospheric delay of the signal path at frequency j; $T_{r}^{s}$ is the tropospheric delay; $d_{r,j}$ and $d_{j}^{s}$ are the code biases of the receiver and the satellite; and $\;e_{r,j}^{s}$ and $\varepsilon_{r,j}^{s}$ denote the sum of measurement noise and multipath error for the pseudorange and carrier phase observations. 

The first order of ionospheric delays can be eliminated by forming a linear combination of observations at different frequencies:

(8) $$\{\begin{array}{c} L_{r,3}^{s}=\rho_{r}^{s}+c\left(dt_{r}-dt^{s}\right)+\lambda_{3}\left(b_{r,3}-b_{3}^{s}\right)+\lambda_{3}N_{r,3}^{s}+T_{r}^{s}+\varepsilon_{r,3}^{s}\\ P_{r,3}^{s}=\rho_{r}^{s}+c\left(dt_{r}-dt^{s}\right)+\lambda_{3}\left(d_{r,3}-d_{3}^{s}\right)+T_{r}^{s}+e_{r,3}^{s}\; \end{array}$$

where $\lambda_{3}=\frac{c}{f_{1}^{2}-f_{2}^{2}}$, $b_{r,3}=f_{1}b_{r,1}-f_{2}b_{r,2}$, $b_{3}^{s}=f_{1}b_{1}^{s}-f_{2}b_{2}^{s}$, $d_{r,3}=f_{1}d_{r,1}-f_{2}d_{r,2}$ and $d_{3}^{s}=f_{1}d_{1}^{s}-f_{2}d_{2}^{s}$; and $f_{1}$ and $f_{2}$ are the frequencies of GPS L1 and L2 measurements. The detailed data processing strategies are shown in Table 2.

### 2.3. Robust Vondrak Filter

The Vondrak filter exhibits several advantages over the other filtering techniques: (1) it does not require a pre-defined fitting function, (2) filter values at the beginning and end of the data series can be calculated, (3) it can cope with unevenly-sampled data, (4) it can be used for separating signals of different frequencies [41]. However, the Vondrak filter also suffers from the limitation that it cannot effectively resist the interference of gross errors. Therefore, we propose an algorithm of the robust Vondrak (R-Vondrak) filter.

The observation sequence of the GPS measuring instantaneous sea levels is denoted as $z\left(t_{i}\right)\left(i=1,2\ldots N\right)$. The basic principle of the Vondrak filter is to derive filter values under the following condition [42]:

(9) $$Q=F+\lambda^{2}S=min$$

with

(10) $$F=\frac{1}{n}\sum_{i=1}^{n}p_{i}{\left[z^{\prime }(t_{i})-z(t_{i})\right]}^{2}$$

(11) $$S=\overset{n-3}{\underset{i=1}{\sum }}{△}^{3}z^{\prime }\left(t_{i}\right)$$

In the above equations, $z^{\prime }\left(t_{i}\right)$ is the filter value corresponding to the measurement sequence $z\left(t_{i}\right)$, $p_{i}$ is the weight of $z\left(t_{i}\right)$, ${△}^{3}z^{\prime }\left(t_{i}\right)$ is the third-order of filter values calculated based on a cubic Lagrange polynomial and $\lambda^{2}$ is a unitless positive coefficient that controls the degree of filtering, i.e., the smoothness of the filtered series.

F is the objective function of the common weighted least squares method, which we call the fitting degree of the smoothing method. S is the quadratic sum of ${△}^{3}z^{\prime }\left(t_{i}\right)$, which reflects the smoothness of the smooth curve in general [42]. When $\lambda^{2}\to \infty$, S→0 and F→min, a smooth parabola can be derived, which is called absolute smoothing. When $\lambda^{2}\to 0$, F→0, the filtering value is approaching the observation value we get.

In order to effectively eliminate the influence of the gross errors, the robust estimation is carried out with the IGG III Scheme [43], and zero weight estimation is used for harmful information. The original weight matrix of the observations is as follows.

(12) P=[p1   p2   ⋱      pn]

The IGG III Scheme for independent observations was based on the following equivalent weight function [43],

(13) $$p_{i}=\{\begin{array}{cc} p_{i} & \left|\tilde{v_{i}}\right|\leq k_{0}\\ p_{i}\frac{k_{0}}{\left|\tilde{v_{i}}\right|}{\left(\frac{k_{1}-\tilde{v_{i}}}{k_{1}-k_{0}}\right)}^{2} & k_{0}<\left|\tilde{v_{i}}\right|\leq k_{1}\\ 0 & k_{1}<\left|\tilde{v_{i}}\right| \end{array}$$

where $\left|\tilde{v_{i}}\right|$ is the standardized residual, $\tilde{v_{i}}=\frac{v_{i}}{\delta_{0}}$,$\delta_{0}=\sqrt{\frac{1}{n}\overset{n}{\underset{i=1}{\sum }}v_{i}^{2}}$; $k_{0}$ and $k_{1}$ are harmonic coefficients, empirically set to 1.0 and 2.5 [43]. As shown in Equation (13), $\left|\tilde{v_{i}}\right|$ is divided into three segments, which represent effective information, available information and harmful information. 

The least squares optimisation can be used to solve Equation (9), in order to obtain the filtered results. The basic principle of least squares is as follows [44]:

(14) $$Q=V^{T}PV=min$$

With

(15) $$V=\left(\begin{array}{c} \frac{1}{\sqrt{n}}E_{n\times n}\\ \frac{\lambda }{\sqrt{n-3}}C_{\left(n-3\right)\times n} \end{array}\right){\hat{Z}}_{n\times 1}-\left(\begin{array}{c} \frac{1}{\sqrt{n}}Z_{n\times 1}\\ 0_{\left(n-3\right)\times 1} \end{array}\right)$$

(16) $$\hat{Z}={\left[z^{\prime }\left(t_{1}\right),z^{\prime }\left(t_{2}\right)\cdots z^{\prime }\left(t_{n}\right)\right]}^{T},\;Z={\left[z\left(t_{1}\right),z\left(t_{2}\right)\cdots z\left(t_{n}\right)\right]}^{T}$$

(17) C(n−3)×n=[−13−3 −131  −31   …  −1   3−31](n−3)×n

(18) $${\left[\begin{array}{c} \frac{1}{\sqrt{n}}E_{n\times n}\\ \frac{\lambda }{\sqrt{n-3}}C_{\left(n-3\right)\times n} \end{array}\right]}^{T}\left[\begin{array}{cc} P_{n\times n} & 0\\ 0 & E_{n\times n} \end{array}\right]\left(\left[\begin{array}{c} \frac{1}{\sqrt{n}}E_{n\times n}\\ \frac{\lambda }{\sqrt{n-3}}C_{\left(n-3\right)\times n} \end{array}\right]{\hat{Z}}_{n\times 1}-\left[\begin{array}{c} \frac{1}{\sqrt{n}}Z_{n\times 1}\\ 0_{\left(n-3\right)\times 1} \end{array}\right]\right)=0$$

$E_{n\times n}$ is the identity matrix. The above Equation (18) can be simplified to Equation (19).

(19) $$\left[P_{n\times n}+\frac{n}{n-3}\lambda^{2}C_{\left(n-3\right)\times n}^{T}C_{\left(n-3\right)\times n}\right]{\hat{Z}}_{n\times 1}=Z_{n\times 1}$$

**Figure 2:**
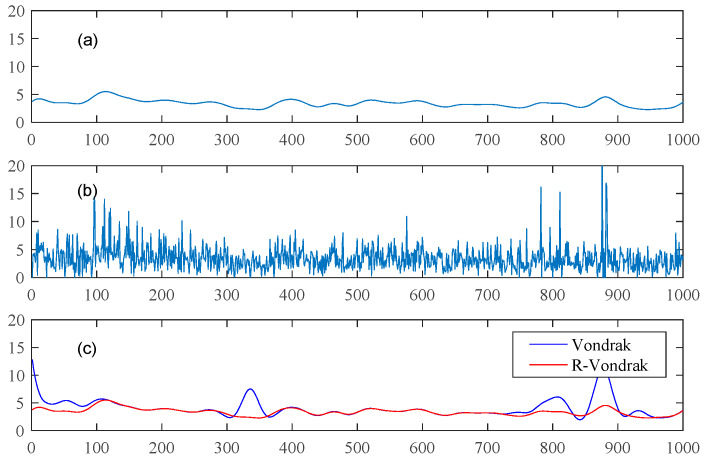
Comparison between the Vondrak filter and the R-Vondrak filter. (**a**) shows the original data; (**b**) shows data after adding random noise and gross errors; (**c**) shows the results of using the Vondrak (blue) and R-Vondrak Filter (red).

Figure 2 shows the results of the Vondrak filter and the R-Vondrak filter. The original data is shown in Figure 2a, Figure 2b shows data after adding random noise and gross errors and Figure 2c shows the results after using these two filtering algorithms. It can be seen that the R-Vondrak filter can effectively eliminate gross errors as compared with the Vondrak filtering algorithm.

## 3. Evaluation of RT-PPP Based on RTS Products

### 3.1. Assessments of the RTS Orbit and Clock Products

In this section, the accuracy of RTS products is assessed through comparison with the IGS final products. We used the BNC software to receive 7-day RTS products from 16 October to 22 October 2017. Taking the IGS final products as reference, the RMS values of the RTS precise orbit and clock bias products were analyzed. 

**Figure 3:**
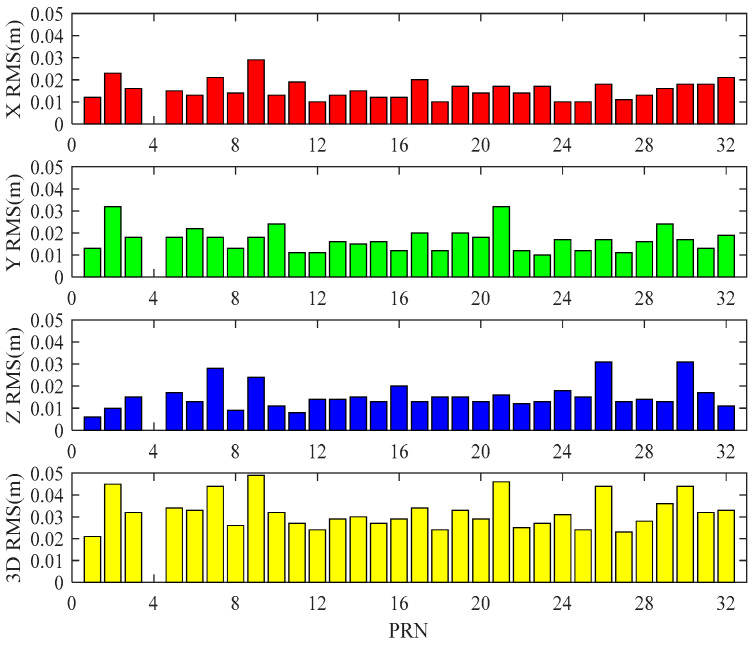
RMS values of corrected satellite orbit using RTS products in X, Y, Z directions and three-dimensional (3D) coordinates.

Figure 3 shows the RMS values of X, Y, Z directions and three-dimensional (3D) coordinates of all satellites corrected by RTS orbit products. It can be found that the RMS values in three directions are less than 0.04 m, and the 3D accuracy is better than 0.05 m, which meet the target accuracy 0.05 m.

**Figure 4:**
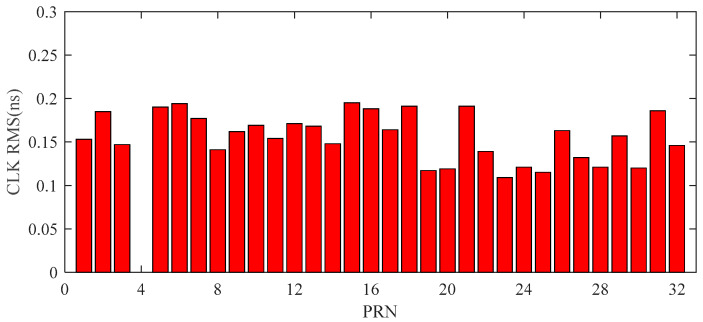
RMS values of corrected satellite clock bias (CLK) using RTS products. CLK is the satellite clock bias, and PRN (Pseudo-Random Noise) stands for the satellite number.

Figure 4 shows the RMS errors of clock bias of all satellites, ranging from 0.13–0.22 ns, with a mean value around 0.15 ns, which is ten-times better than the predicted part of the ultra-rapid clock bias (shown in Table 1) and meets the target accuracy 0.3 ns.

### 3.2. Performance of RT-PPP Using the RTS Products

**Figure 5:**
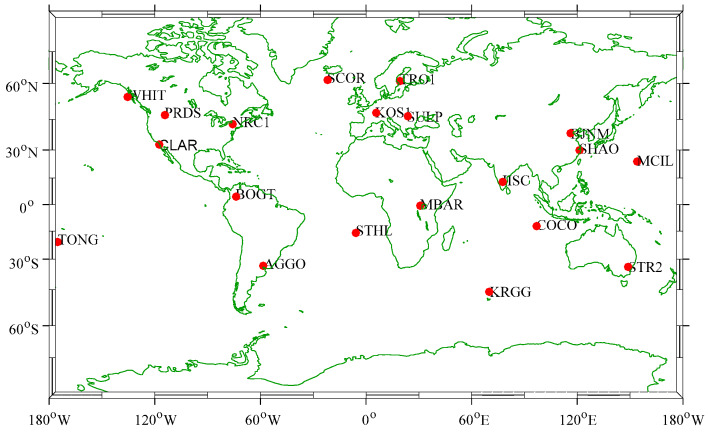
20 IGS stations around the world for kinematic RT-PPP positioning assessment.

In the kinematic positioning mode, we used GPS data collected from 16 October to 22 October 2017 in 20 IGS stations around the world to evaluate the performance of RT-PPP. Meanwhile, the IGS final (IGS) products and IGS ultra-rapid (IGU) are used for comparison. Locations of the 20 stations are illustrated in Figure 5. The specific processing strategies of PPP are shown in Table 2.

**Figure 6:**
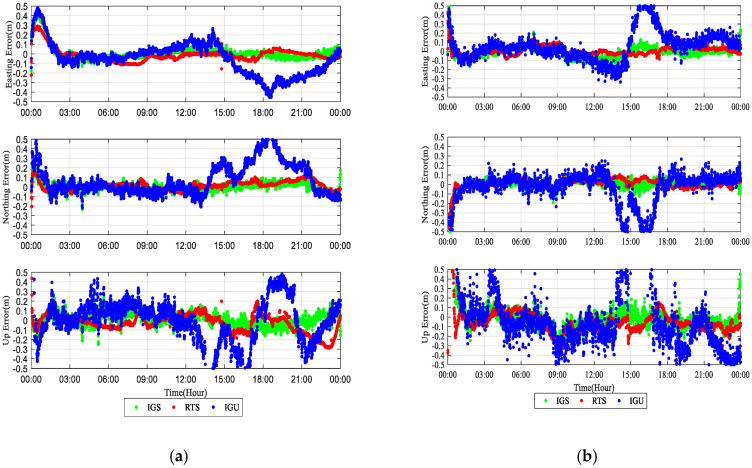
Kinematic precise point positioning (PPP) solutions using the RTS products (red), the IGS ultra-rapid (IGU) products (blue) and the IGS products (green) at station KOS1 (**a**) and SHAO (**b**) on 19 October 2017.

Figure 6 shows the kinematic position results of the three products on 19 October 2017 in KOS1 (52.17° N, 5.82° E) and SHAO (31.10° N, 121.20° E). It can be seen that the RTS products have a higher positioning accuracy and stability as compared to the IGU products, as well as good consistency with post-processing PPP using the IGS products.

**Figure 7:**
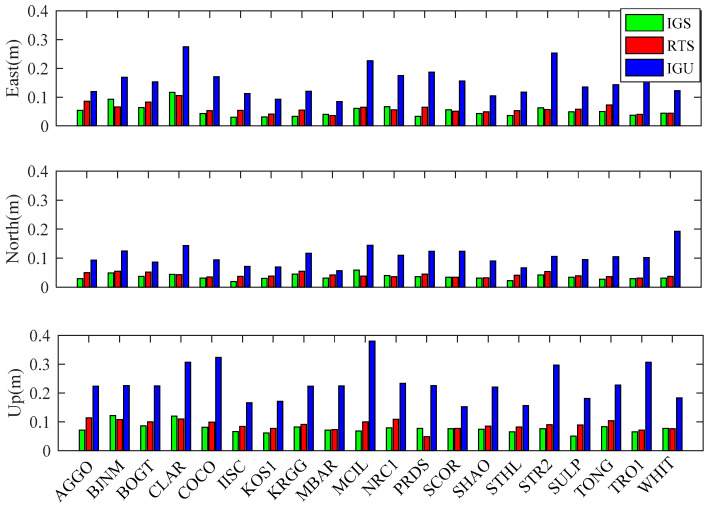
Average RMS of kinematic PPP solutions at 20 IGS stations from 16 October to 22 October 2017.

Figure 7 illustrates the RMS averaged from 7 days at each of 20 stations in the kinematic position mode. As expected, the performance of the IGS products is the best due to its high accuracy and stability. In the two real-time products, the average RMS value of the RTS products is better than that of the IGU products. In the east coordinate, a reduction in the RMS values from 0.153 to 0.059 m can be observed for the RT-PPP solution using the RTS products, as compared to the solution using the IGU products. In the north coordinate, the value is 0.041 for the RTS products, as compared to 0.106 for the IGU products. Moreover, the accuracy of the vertical component is 0.087 m for the RTS products and 0.232 m for the IGU products. Overall, average improvements of 61.4%, 61.3% and 62.5% are achieved by RT-PPP using the RTS products compared to the IGU products.

In general, due to the precise satellite orbit and clock corrections, performance of the RTS products is better than the IGU products, especially in the vertical direction, which shows a good stability and accuracy in kinematic positioning mode. Therefore, the RTS products are more suitable for the real-time tide measurement.

## 4. Experiment and Data Analysis

**Figure 8:**
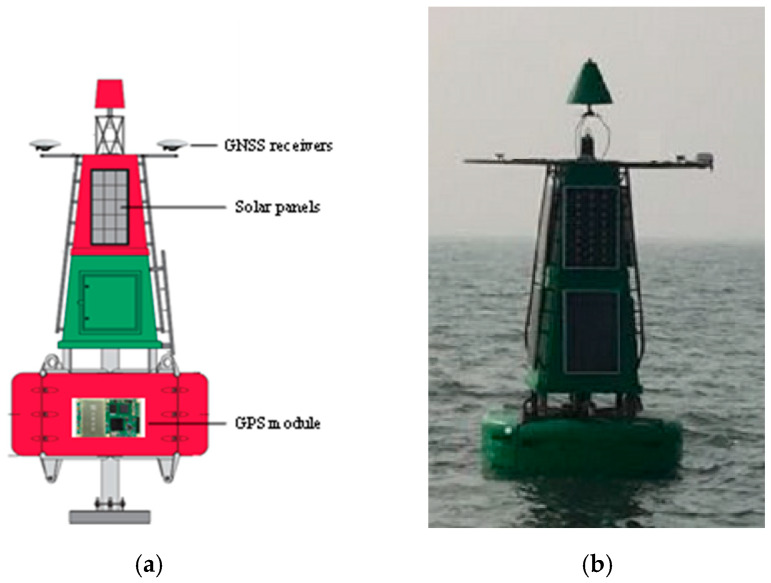
Structure of the multi-functional buoy. (**a**) Shows installation position of GPS receivers, GPS module and solar panels; (**b**) shows the buoy at the testing field. GNSS: global navigation satellite system.

In order to adapt to the different sea conditions, a multi-functional buoy with solar cells was used in this experiment. As shown in Figure 8, this buoy had a diameter of 2.5 m, a height of 5 m and a mass of about 1.5 t, which can achieve good stability under different sea conditions and provide power support for the buoy system. Real-time tide information can be observed by installing GPS and other equipment on the buoy. In this experiment, the Sinan K528 dual-frequency positioning module with dual antenna was used to provide real-time positioning information. The structure of the buoy and the installation of the GPS module are shown in Figure 8a, and Figure 8b shows the buoy at the testing field.

**Figure 9:**
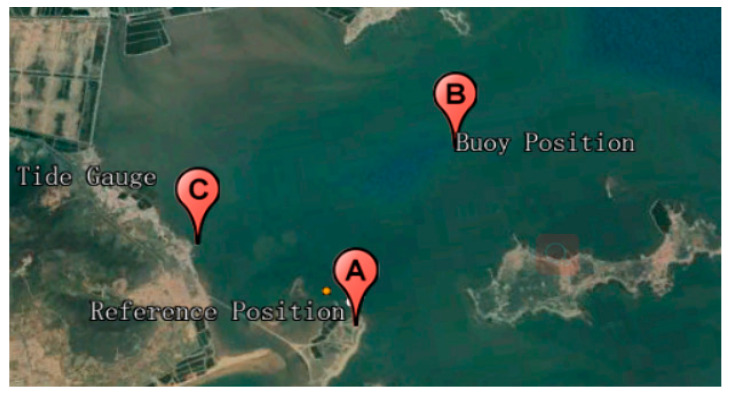
The location of study area near Qingdao, China. A is the fixed reference GPS station, B is the multi-function buoy, and C is the long-term tide gauge station.

Figure 9 shows the field experiment in the Yellow Sea of China near Qingdao. The buoy was deployed at position B, covering about 22 h from 0:00:00 to 21:59:59 (Coordinated Universal Time, UTC) on 19 October 2017. To assess the accuracy of real-time tide measurements using the RT-PPP method, the results were evaluated by comparing with the post-processing PPP method, the double difference (DD) method and nearby tide gauge data. Therefore, we installed a fixed-reference GPS station at point A, about 3 km away from the buoy. A KPTKQ4EE22O NET-G3A receiver and Topcon TPSCR.G3 antenna were used in the reference station, and coordinates were determined by the average of the daily PPP solution on 19 October 2017. Point C was the long-term tide gauge station, which was about 2 km away from the buoy, and the data was converted to ITRF 2014. The GPS signals were recorded at 1 Hz at the buoys and the reference station, and the sampling rate of the tide gauge station was 10 min. Meanwhile, the RTS products were received with an interval of 5 s for real-time correction of broadcast ephemeris and clock bias.

**Figure 10:**
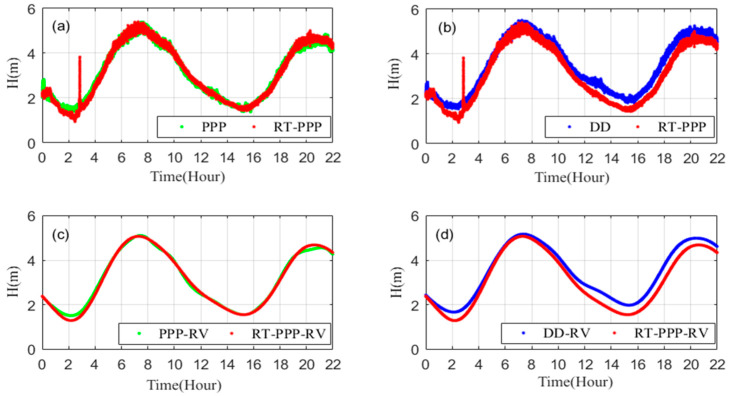
Comparison of RT-PPP tide measurement results (red) with the post-processing PPP method (green) and double difference (DD) method (blue). (**a**,**b**) Show the instantaneous sea level, (**c**,**d**) show filtered tide values.

Figure 10 shows the instantaneous sea level and filtered tide value measured by RT-PPP based on the RTS products. We compared the results with post-processing PPP and DD methods—the specific results are shown in Table 3. Compared with post-processing PPP and DD, the average biases of RT-PPP are 0.090 m and 0.181 m, respectively, and the RMS values are 0.128 m and 0.223 m, respectively. From Figure 10a,b we see that gross errors occur at about 3:00:00 due to the loss of RTS correction data, combined with Figure 10c,d we can find that the R-Vondrak filtering algorithm can eliminate the gross errors and high-frequency noise effectively. After filtering, the maximum bias decreased from 2.231 m and 2.268 m to 0.225 and 0.447 m, and the RMS values are 0.090 m and 0.203 m in comparison to the post-processing PPP method and the DD method.

**Figure 11:**
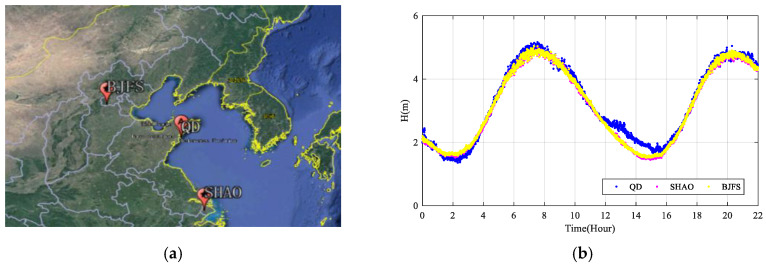
The instantaneous sea level comparation of the DD method using three different base stations. (**a**) Shows IGS stations BJFS, SHAO and our reference station QD, (**b**) shows the three DD results without outlies of the instantaneous sea level with different base stations.

As can be noted, the RT-PPP results are in good agreement with the post-processing PPP tide measurement method. However, we can see an obvious difference between the RT-PPP tide measurement method and the DD method in Figure 10 and Table 3. We assume that this phenomenon may be due to the influence of the base station, such as the multi-path effect on the shore and the inaccurate coordinates. In order to test our hypothesis, we selected two IGS stations, BJFS and SHAO (shown in Figure 11a) which are close to the buoy in contrast to others, with a coordinate accuracy at millimeter level. We used these two stations as base stations respectively to obtain the instantaneous sea level, and we compared the DD measurement results with our own reference station (QD), which is shown in Figure 11b. Table 4 shows the cross-validated results of the instantaneous sea level height based on these three reference stations. We can find a significant difference between the DD results of QD and BJFS/SHAO—it seems that there is a problem with QD base receiver/antenna. In the DD tide measurement, the precision of tide data is highly dependent on the precision and reliability of the base station.

**Figure 12:**
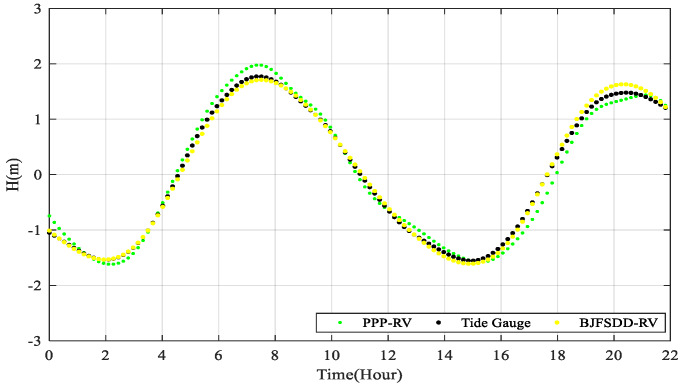
Comparison of the DD tide based on the BJFS and PPP tide with tide gauge data.

In Figure 12, we evaluate the DD tide and post-processing PPP tide respectively based on the data of tide gauge, and specific results are shown in Table 5. We can see that the average bias of BJFS-DD tide is 0.053 m, and RMS value is 0.067 m, which are 2-times better than the PPP tide. Due to the problem of the QD station, the DD tide based on BJFS is selected as the reference DD solution.

**Figure 13:**
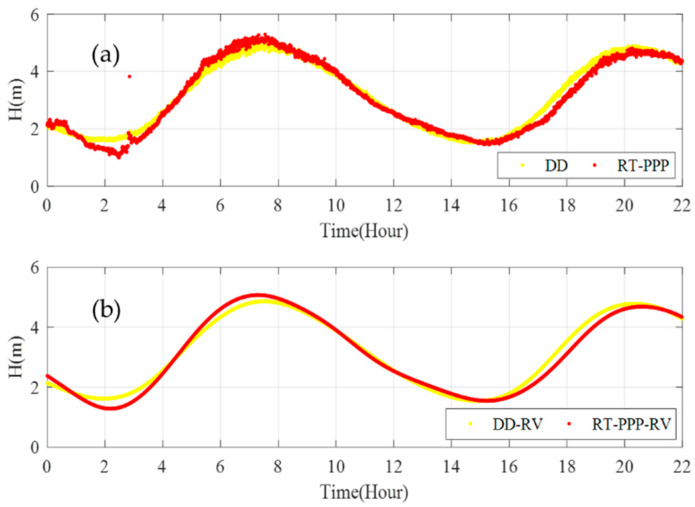
Comparison of the RT-PPP tide measurement results with the DD method based on BJFS. (**a**) Shows the instantaneous sea level, (**b**) shows the filtered tide values.

In Figure 13, we compare the measurement results of RT-PPP with the DD method based on BJFS, and the specific results are shown in Table 6. The RMS value of the instantaneous sea level measured by RT-PPP is 0.213 m, and the RMS value of the tide data is 0.194 m after filtering.

**Figure 14:**
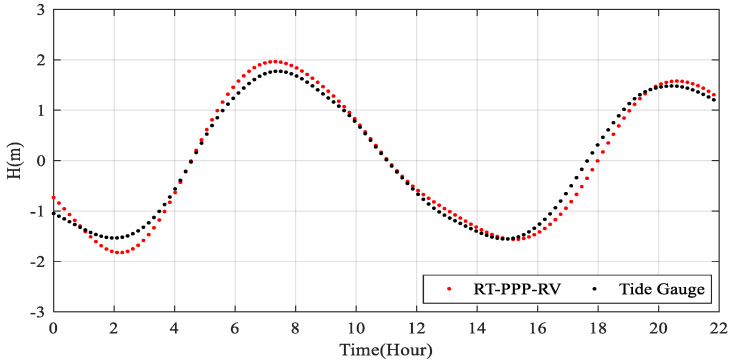
The RT-PPP tide measurement results with respect to the tide gauge data.

The reliability of the RT-PPP for tide measurement is further verified by the long-term tide gauge nearby, which is shown in Figure 14. As the sampling rate of the tide gauge is 10 min in contrast to 1 s of the GPS receiver on the buoy, we extract the buoy data at the measurement time of tide gauge. Table 7 illustrates the average bias and RMS value between the RT-PPP results and the tide gauge data, which are 0.141 m and 0.167 m.

## 5. Discussion

This paper describes a method of RT-PPP tide measurement based on RTS products, which is different from the traditional method of using tide gauges, satellite altimeters and GNSS-R measurement. To eliminate the gross errors and high frequency fluctuations caused by wind and waves, we propose an algorithm of a robust Vondrak filter.

From the experimental results in the Yellow Sea of China near Qingdao, we can see the performance of the RT-PPP tide measurement algorithm. The results of the real-time tide measurement achieved with the RT-PPP method is investigated through comparison and validation with the post-processing PPP method, the DD method and the tide gauge data nearby. Good consistency can be found with post-processing PPP method and the tide gauge data. However, an obvious difference between the RT-PPP tide measurement method and the DD method can be found due to the problem of the QD reference station. The DD tide measurement has a limitation that it relies on the precision and stability of the reference station.

**Figure 15:**
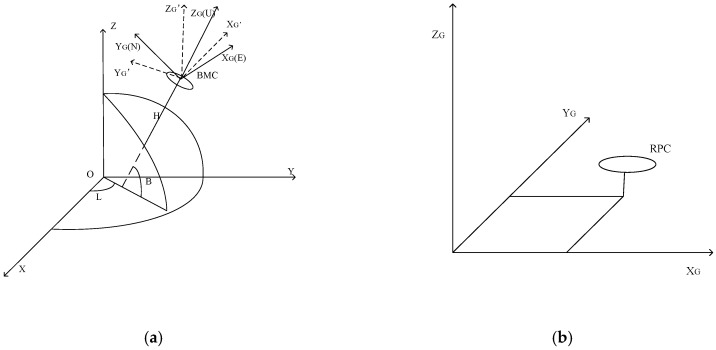
Illustration of the reason for attitude errors. (**a**) Shows the vertical direction of the carrier coordinate system which is inconsistent with that of the ellipsoid geodetic coordinate system due to the buoy attitude changing, (**b**) shows coordinates of the antenna phase center in carrier coordinate system. RPC: receiver phase center, BMC: buoy mass center.

In the RT-PPP tide measurement, although the results are in good agreement with the post-processing PPP method and tide gauge data, there is still a certain gap in the accuracy compared with the kinematic solution results of the IGS stations shown in Figure 7, and the possible reasons are as follows:

(1) When the GPS buoy is used for tide measurement, the attitude of the buoy suffers from fluctuations due to the influence of wind, waves and other factors. As shown in Figure 15a, the vertical direction of the carrier coordinate system with buoy mass center (BMC) as the origin is inconsistent with that of the ellipsoid geodetic coordinate system. The receiver phase center (RPC) is usually not consistent with the BMC, which is shown in Figure 15b, resulting in errors in a vertical direction. If the attitude correction is not considered, system errors might occur under the severe sea conditions. 

During the tide measurement, the buoy produces attitude changes in pitch, roll and heading direction. The coordinates of RPC in the carrier coordinate system are X, Y and Z, and the corrected coordinates $X^{\prime }$, $Y^{\prime }$ and $z^{\prime }$ are calculated as the specific method in reference [45].

The geodetic coordinates of the antenna phase center measured by GPS are B, L and H. The geodetic height of the sea surface $H_{r}$ is calculated as follows:

(20) $$H_{r}=H-Z^{\prime }-h^{\prime }+{h_{r}}^{\prime }$$

where $h^{\prime }$ is the height h from the BMC to the bottom of the buoy after correction and ${h_{r}}^{\prime }$ is the heave of the buoy $h_{r}$ after correction. h and $h_{r}$ need to be calibrated before the buoy is used on the sea.

(2) The sea surface is rough, and some GPS signals from multiple satellites and multiple directions will be reflected by the sea surface to the GPS antenna. Multipath disturbance is one of the most important error sources in high-accuracy positioning and navigation.

(3) Although the accuracy of the RTS products is better than the IGU products, the positioning accuracy of the RT-PPP is limited in comparison to the post-processing PPP method, especially in the vertical direction, which is about 0.1 m.

In future research, the accuracy of real-time tide measurement needs to be further improved. Firstly, attitude correction needs to be used to adapt to different sea conditions. Then, we need to eliminate the effects of multipath GPS reflection signal, such as selecting a brand of GPS antenna that is resistant to multipath effects and using choke-ring antennas. Finally, with the development of Beidou, GLONASS, Galileo and other GNSS signals, further research should focus on multi-GNSS signals combinations which can improve positioning accuracy and reliability.

## 6. Conclusions

This research aimed to provide continuous real-time tide data in different sea areas. For this purpose, a detailed description of the real-time tide measurement method based on RT-PPP was introduced. To validate the performance of RT-PPP-based tide measurement, firstly, the qualities of RTS orbit and clock corrections over a 7-day testing period were investigated by taking IGS final products as a reference. Secondly, 7-day observation data from 20 globally distributed stations in the IGS network was used to assess the kinematic position accuracy of RT-PPP using RTS products. Finally, the reliability of the RT-PPP-based tide was investigated through comparison and validation with other three scenarios, i.e., the post-processing PPP method, the DD method and the nearby tide gauge data.

The evaluation results indicate that the orbit and clock corrections of RTS products are better than 0.05 m for orbits, and 0.22 ns less for clock bias, respectively. Additionally, the average RMS values of the kinematic PPP in east, north and up directions by using RTS products are 0.059 m, 0.041 m and 0.087 m, respectively, with a significant improvement of about 60% when compared to using IGU products. For the performance of RT-PPP-based tide measurement, the experimental results in the Yellow Sea of China show that, compared with the other three tide measurement methods, the average bias of RT-PPP tide measurement are 0.065 m, 0.194 m and 0.167 m, and the RMS values are 0.090 m, 0.194 m and 0.167 m, respectively, indicating that the accuracy of RT-PPP tide measurement can be achieved at sub-decimeter level.

Our proposed method has shown the great potential in applications for continuous real-time tide observation and sea level monitoring in different sea areas. For our future work, we will focus on other challenges in improving the accuracy of real-time tide measurements, e.g., attitude correction, reducing the impact of multi-path effect on the sea and using multi-GNSS combinations.

## Figures and Tables

**Table 1:** International GNSS Service (IGS) ultra-rapid and final products description.

Product	GPS Orbit/Clock	Accuracy	Latency	Interval
Ultra-rapid (predicted part)	Orbit Satellite clocks	5 cm 3 ns RMS	Real time	15 min
Final	Orbit Satellite clocks	2.5 cm 75 ps RMS	12–18 days	15 min 30 s

**Table 2:** Data processing strategies of RT-PPP.

Item	Strategies
Observations	Undifferenced phase and code observations
Observation model	Ionospheric-free linear combinations
Frequency selection	Global positioning system (GPS): L1 and L2
Elevation cutoff angle	10°
Weighing strategy	Elevation dependent weight
Receiver phase center	Phase center offset (PCO) and phase center variation (PCV) values from igs08.atx
Satellite phase center	PCO and PCV values from igs08.atx
Phase-windup effect	Model corrected
Zenith tropospheric delay	A priori value provided by the UNB3m+global mapping function (GMF)

**Table 3:** Comparison of RT-PPP tide measurement results before and after filtering with the post-processing PPP method and the DD method.

Compared Methods	Before Filtering			After Filtering		
	Bias(m)		RMS(m)	Bias(m)		RMS(m)
	Max	Average		Max	Average	
RT-PPP - PPP	2.231	0.090	0.128	0.225	0.065	0.090
RT-PPP - DD	2.268	0.181	0.223	0.447	0.166	0.203

**Table 4:** Cross-validation of DD instantaneous sea level using three different base stations.

Base Stations	Bias(m)		RMS(m)
	Max	Average	
QD - SHAO	0.681	0.134	0.180
QD - BJFS	0.678	0.126	0.172
BJFS - SHAO	0.110	0.028	0.035

**Table 5:** Accuracy of the DD tide based on the BJFS and PPP tide with reference to tide gauge data.

Compared Methods	Bias(m)		RMS(m)
	Max	Average	
BJFSDD - Tide	0.156	0.053	0.067
PPP - Tide	0.333	0.118	0.139

**Table 6:** Comparison of the RT-PPP tide measurement results with the DD method based on BJFS.

Compared Methods	Before Filtering			After Filtering		
	Bias(m)		RMS(m)	Bias(m)		RMS(m)
	Max	Average		Max	Average	
RT-PPP - DD	2.028	0.166	0.213	0.415	0.155	0.194

**Table 7:** Comparison of the RT-PPP tide measurement results with the tide gauge data.

Compared Methods	Bias(m)		RMS(m)
	Max	Average	
RT-PPP - Tide	0.343	0.141	0.167

## References

[1] Douglas B.C. (2001). Sea level change in the era of the recording tide gauge. International Geophysics.

[2] Menendez M., Woodworth P.L. (2010). Changes in extreme high water levels based on a quasi-global tide-gauge data set. J. Geophys. Res. Space Phys..

[3] Church J.A., White N.J. (2011). Sea-level rise from the late 19th to the Early 21st Century. Surv. Geophys..

[4] Kopp R.E., Horton R.M., Little C.M., Mitrovica J.X., Oppenheimer M., Rasmussen D.J., Strauss B.H., Tebaldi C. (2014). Probabilistic 21st and 22nd century sea-level projections at a global network of tide-gauge sites. Earth’s Future.

[5] Arabelos D., Papazachariou D., Contadakis M., Spatalas S. (2011). A new tide model for the Mediterranean Sea based on altimetry and tide gauge assimilation. Ocean Sci..

[6] Gharineiat Z., Deng X. (2020). Spectral analysis of satellite altimeter and tide gauge data around the Northern Australian Coast. Remote Sens..

[7] Merrifield M., Firing Y., Aarup T., Agricole W., Brundrit G., Chang-Seng D., Farre R., Kilonsky B., Knight W., Kong L. (2005). Tide gauge observations of the Indian Ocean tsunami, December 26, 2004. Geophys. Res. Lett..

[8] Rabinovich A.B., Thomson R.E. (2007). The 26 December 2004 Sumatra tsunami: Analysis of tide gauge data from the world ocean Part 1. Indian Ocean and South Africa. Tsunami and Its Hazards in the Indian and Pacific Oceans.

[9] Fujii Y., Satake K. (2007). Tsunami source of the 2004 Sumatra–Andaman earthquake inferred from tide gauge and satellite data. Bull. Seismol. Soc. Am..

[10] Ray R.D. (1999). A Global Ocean Tide Model from TOPEX/POSEIDON Altimetry: GOT99. 2.

[11] Watson C., Coleman R., White N., Church J., Govind R. (2003). Absolute calibration of TOPEX/Poseidon and Jason-1 using GPS buoys in bass strait, Australia special issue: Jason-1 calibration/validation. Mar. Geod..

[12] Bonnefond P., Exertier P., Laurain O., Ménard Y., Orsoni A., Jan G., Jeansou E. (2003). Absolute calibration of Jason-1 and TOPEX/Poseidon altimeters in Corsica special issue: Jason-1 calibration/validation. Mar. Geod..

[13] Bonnefond P., Exertier P., Laurain O., Ménard Y., Orsoni A., Jeansou E., Haines B.J., Kubitschek D.G., Born G. (2003). Leveling the sea surface using a GPS-catamaran special issue: Jason-1 calibration/validation. Mar. Geod..

[14] Vu P., Frappart F., Darrozes J., Marieu V., Blarel F., Ramillien G., Bonnefond P., Birol F. (2018). Multi-satellite altimeter validation along the french atlantic coast in the southern bay of biscay from ers-2 to saral. Remote Sens..

[15] Martin-Neira M. (1993). A passive reflectometry and interferometry system (PARIS): Application to ocean altimetry. ESA J..

[16] Treuhaft R.N., Lowe S.T., Zuffada C., Chao Y. (2001). 2-cm GPS altimetry over Crater Lake. Geophys. Res. Lett..

[17] Löfgren J.S., Haas R., Johansson J.M. (2011). Monitoring coastal sea level using reflected GNSS signals. Adv. Space Res..

[18] Santamaría-Gómez A., Watson C., Gravelle M., King M., Wöppelmann G. (2015). Levelling co-located GNSS and tide gauge stations using GNSS reflectometry. J. Geod..

[19] Jin S., Qian X., Wu X. (2017). Sea level change from BeiDou Navigation Satellite System-Reflectometry (BDS-R): First results and evaluation. Glob. Planet. Chang..

[20] Martin-Neira M., Caparrini M., Font-Rossello J., Lannelongue S., Vallmitjana C.S. (2001). The PARIS concept: An experimental demonstration of sea surface altimetry using GPS reflected signals. IEEE Trans. Geosci. Remote Sens..

[21] Lowe S.T., Kroger P., Franklin G., LaBrecque J.L., Lerma J., Lough M., Marcin M.R., Muellerschoen R.J., Spitzmesser D., Young L.E. (2002). A delay/Doppler-mapping receiver system for GPS-reflection remote sensing. IEEE Trans. Geosci. Remote Sens..

[22] Ruffini G., Soulat F., Caparrini M., Germain O., Martín-Neira M. (2004). The eddy experiment: Accurate GNSS-R ocean altimetry from low altitude aircraft. Geophys. Res. Lett..

[23] Kato T., Terada Y., Kinoshita M., Kakimoto H., Isshiki H., Matsuishi M., Yokoyama A., Tanno T. (2000). Real-time observation of tsunami by RTK-GPS. Earth Planets Space.

[24] Watson C., Coleman R., Handsworth R. (2008). Coastal tide gauge calibration: A case study at Macquarie Island using GPS buoy techniques. J. Coast. Res..

[25] Bouin M.-N., Ballu V., Calmant S., Pelletier B. (2009). Improving resolution and accuracy of mean sea surface from kinematic GPS, Vanuatu subduction zone. J. Geod..

[26] Apel H., Hung N.G., Thoss H., Schöne T. (2012). GPS buoys for stage monitoring of large rivers. J. Hydrol..

[27] Zumberge J., Heflin M., Jefferson D., Watkins M., Webb F. (1997). Precise point positioning for the efficient and robust analysis of GPS data from large networks. J. Geophys. Res. Solid Earth.

[28] Blewitt G., Kreemer C., Hammond W.C., Plag H.P., Stein S., Okal E. (2006). Rapid determination of earthquake magnitude using GPS for tsunami warning systems. Geophys. Res. Lett..

[29] Li X., Ge M., Zhang H., Wickert J. (2013). A method for improving uncalibrated phase delay estimation and ambiguity-fixing in real-time precise point positioning. J. Geod..

[30] Guo B., Zhang X., Ren X., Li X. (2015). High-precision coseismic displacement estimation with a single-frequency GPS receiver. Geophys. J. Int..

[31] Liu R., Guo B., Zhang A., Yimwadsana B. (2020). Research on GPS precise point positioning algorithm with a Sea Surface Height Constraint. Ocean Eng..

[32] IGS IGS Quality of Service Fact Sheet. https://kb.igs.org/hc/en-us/articles/201208216-IGS-Quality-of-Service-Fact-Sheet.

[33] Wang J., He X., Ferreira V. (2015). Ocean wave separation using CEEMD-Wavelet in GPS wave measurement. Sensors.

[34] Weber G., Mervart L., Lukes Z., Rocken C., Dousa J. Real-time clock and orbit corrections for improved point positioning via NTRIP. Proceedings of the ION-GNSS-2007, Institute of Navigation.

[35] Liu T., Yuan Y., Zhang B., Wang N., Tan B., Chen Y. (2017). Multi-GNSS precise point positioning (MGPPP) using raw observations. J. Geod..

[36] Li X., Ge M., Zhang X., Zhang Y., Guo B., Wang R., Klotz J., Wickert J. (2013). Real-time high-rate co-seismic displacement from ambiguity-fixed precise point positioning: Application to earthquake early warning. Geophys. Res. Lett..

[37] Lu C., Li X., Ge M., Heinkelmann R., Nilsson T., Soja B., Dick G., Schuh H. (2016). Estimation and evaluation of real-time precipitable water vapor from GLONASS and GPS. GPS Solut..

[38] Weber G., Mervart L. (2012). BKG Ntrip Client (BNC) Version 2.7 Manual, Federal Agency for Cartography and Geodesy.

[39] Li X., Ge M., Dai X., Ren X., Fritsche M., Wickert J., Schuh H. (2015). Accuracy and reliability of multi-GNSS real-time precise positioning: GPS, GLONASS, BeiDou, and Galileo. J. Geod..

[40] Ge M., Gendt G., Rothacher M., Shi C., Liu J. (2008). Resolution of GPS carrier-phase ambiguities in precise point positioning (PPP) with daily observations. J. Geod..

[41] Simon D. (2001). Kalman filtering. Embed. Syst. Program..

[42] Vondrák J. (1977). Problem of smoothing observational data II. Bull. Astron. Inst. Czechoslov..

[43] Yang Y. (1994). Robust estimation for dependent observations. Manuscr. Geod..

[44] Tao T., Liu J., Qu X., Gao F. (2019). Real-time monitoring rapid ground subsidence using GNSS and Vondrak filter. Acta Geophys..

[45] Lu G. (1995). Development of a GPS Multi-Antenna System for Attitude Determination.

